# Real-world treatment of German patients with recurrent and advanced endometrial cancer with a post-platinum treatment: a retrospective claims data analysis

**DOI:** 10.1007/s00432-022-04183-y

**Published:** 2022-07-16

**Authors:** Antje Mevius, Florian Karl, Margarethe Wacker, Robert Welte, Stefanie Krenzer, Theresa Link, Ulf Maywald, Thomas Wilke

**Affiliations:** 1 IPAM e.V, University of Wismar, Wismar, Germany; 2 GSK, Munich, Germany; 3 Department of Gynecology and Obstetrics, Technische Universität Dresden, Dresden, Germany; 4 National Center for Tumor Diseases (NCT/UCC), Dresden, Germany; 5 German Cancer Research Center (DKFZ), Heidelberg, Germany; 6 Faculty of Medicine and University Hospital Carl Gustav Carus, Technische Universität Dresden, Dresden, Germany; 7 Helmholtz-Zentrum Dresden-Rossendorf (HZDR), Dresden, Germany; 8 AOK PLUS, Dresden, Germany

**Keywords:** Endometrial cancer, Post-platinum therapy, Overall survival, Claims data analysis, Retrospective analysis studies

## Abstract

**Purpose:**

Endometrial cancer (EC) is the sixth most common malignancy among females worldwide. Due to limited therapeutic options, treatment of advanced or recurrent disease is associated with poor outcomes. The aim of this study was to describe the real-world treatment of patients with advanced or recurrent EC who received a systemic treatment following platinum-based chemotherapy.

**Methods:**

This retrospective cohort study was based on anonymized German claims data covering the period between January 1, 2010, and June 30, 2020. Patients with EC who started an anticancer treatment following platinum-based chemotherapy were observed for a minimum follow-up of 12 months. Available claims data were used to describe patient characteristics, subsequent treatment lines, healthcare resource utilization, and overall survival (OS) of patients.

**Results:**

Out of 713 patients with advanced or recurrent EC and who had received a platinum-based treatment, 201 (mean age: 68.9 years) with a post-platinum-based treatment were identified and observed. The median OS in this population was 335.0 days. Of the 201 patients, 79 patients (39.3%) received a second line of treatment (LOT), and 21 patients (10.4%) had 3 or more treatment lines. In the LOTs following platinum-based chemotherapy, more than 70 different treatment regimens were observed. The hospitalization rate was generally high, with 5.2 hospitalizations per patient-year in the follow-up period.

**Conclusion:**

The wide variety of therapeutic regimens applied in patients in Germany who progressed after platinum-based therapy confirms the lack of therapeutic strategy for these patients, and the poor prognosis highlights the urgent need for new treatment strategies.

**Supplementary Information:**

The online version contains supplementary material available at 10.1007/s00432-022-04183-y.

## Introduction

Endometrial cancer (EC) is the sixth most common malignancy among women worldwide, with an estimated incidence of about 417,367 new cases globally (World Health Organization 2020b). In Germany, 12,356 newly diagnosed cases were reported in 2020 (World Health Organization 2020a).

More than 90% of diagnosed women are postmenopausal, with a median age at diagnosis of 63 years (Colombo et al. 2016). Multiple risk factors have been reported for EC, most commonly obesity, long-lasting endogenous or exogenous hyperestrogenism (polycystic ovary, tamoxifen therapy, anovulation, nulliparity), hypertension, and diabetes mellitus (Arnold et al. 2015; Colombo et al. 2016; Raglan et al. 2019). In addition, up to 5% of ECs are associated with Lynch syndrome type II (known as hereditary nonpolyposis colorectal carcinoma syndrome), and those with this syndrome have a lifetime risk of 30–60% for developing EC (Colombo et al. 2016).

EC is generally staged according to the International Federation of Gynecology and Obstetrics (FIGO) system (Edey and Murdoch 2010; Pecorelli 2009). Five-year survival rates for EC vary depending on the stage at diagnosis, with approximately 70% of EC cases diagnosed at an early stage. For stage I and II tumors, a 5-year survival rate of more than 95% has been reported (Bock et al. 2018, Yen et al. 2020). However, reported survival rates decrease dramatically for patients with advanced (stage III/IV) EC. In Germany, the overall 5-year survival rate for patients with EC is approximately 78% (Zentrum für Krebsregisterdaten im Robert Koch-Institut 2016). According to the European Society for Medical Oncology, European Society for Radiotherapy and Oncology, and European Society of Gynaecological Oncology Consensus Conference on Endometrial Cancer held in 2014, the 5-year survival rate for patients with stage III EC is 68%, and only 17% for patients with stage IV EC (Colombo et al. 2016).

As described in German and European guidelines on EC (Colombo et al. 2016; Concin et al. 2021; Emons and Steiner 2018), women with advanced or recurrent EC have limited treatment options. Available German treatment guidelines recommend surgery for early-stage EC and adjuvant chemotherapy and radiation for advanced disease. For recurrent disease, hormone therapy is considered and systemic chemotherapy recommended. The guidelines, however, do not specify which chemotherapy to use and, indeed, highlight that there is a lack of evidence on their comparative effectiveness or even the best supportive care (Emons and Steiner 2018). Furthermore, there are no recommendations for second-line treatment of advanced or recurrent EC.

Therefore, it is important to know how patients with advanced or recurrent EC in a post-platinum setting are treated in the real world, and what outcomes are associated with these treatments. This study aimed to describe the real-world treatment of patients with advanced or recurrent EC who had initiated an anticancer treatment following platinum-based chemotherapy as well as the healthcare resource utilization (HCRU) and overall survival (OS) of these patients.

## Methods

### Study design

This retrospective cohort study was based on anonymized claims data covering the period from January 1, 2010, to June 30, 2020, provided by the German statutory health insurance fund AOK PLUS. This dataset covers approximately 3.4 million individuals from the German federal states of Saxony and Thuringia, representing around 4% of the German population.

German claims data provide information on patients’ demographics (age, gender, date of death) and detailed reimbursement claims on outpatient care, inpatient care, pharmaceutical treatments, therapeutic devices, rehabilitation, and sick leave.

Outpatient care data comprise information on diagnostic and therapeutic procedures (according to the German Uniform Valuation Scheme [EBM]) (Kassenärztliche Bundesvereinigung 2021), the diagnosis made by an outpatient physician, and the type of treating physician (identified by the physician code, the “Arztgruppenschlüssel” [AGS]). Inpatient care data cover information on the date of admission and discharge, diagnostic and therapeutic procedures (according to the operation and procedure coding, the “Operationen- und Prozedurenschlüssel” [OPS]) (BfArM 2021). Inpatient and outpatient diagnoses are coded according to the German Modification of the International Classification of Diseases 10th Revision (ICD-10-GM). Data on outpatient prescriptions of reimbursed drugs include information on the date of prescription, the type of prescribing physician, and the pharmaceutical reference number (PZN) of the prescribed agents. The PZN is linked to information on the Anatomical Therapeutic Chemical (ATC) classification code (Fricke 2019), the defined daily dose (DDD), the packaging size, the strength, and the formulation of the drug.

### Study population

Insured females were identified as prevalent EC cases if at least one inpatient EC diagnosis (ICD-10-GM code C54.-, excluding C54.2 [malignant neoplasms of myometrium]) or two confirmed outpatient EC diagnoses made by a specialist (gynecologist, oncologist) were recorded between January 1, 2010, and June 30, 2019. The analysis sample considered identified prevalent patients with EC who received platinum-based therapy (identified via ATC code L01XA- or OPS 8–54) and then initiated a second regimen, and who did not receive any platinum-based treatment in the preceding 12 months (platinum-based treatment-free period); this period was chosen to identify patients with EC who received their first platinum-based treatment. Based on current treatment guidelines recommending chemotherapy only for patients with advanced disease, all patients who had received platinum-based therapy were considered to have advanced stage disease (Colombo et al. 2016; Concin et al. 2021; Emons and Steiner 2018). In addition, to ensure the platinum-based treatment observed was related to EC, only cases with a confirmed EC diagnosis documented 14 days before, or at the latest 3 months after, the platinum-based therapy was started were considered. Finally, patients with an anticancer treatment following the platinum-based chemotherapy were selected. Only anticancer treatments (definition of EC-related treatments and identification codes are provided in Supplemental Table 1) that were initiated more than 3 months after the platinum-based therapy was started were considered as post-platinum-based therapy. The first application/prescription date of the treatment following the platinum-based chemotherapy was defined as the index date.

**Table 1:** Baseline characteristics of identified patients with EC initiating an anticancer treatment following a platinum-based therapy

	Identified EC patient starting a post-platinum-based therapy
*N*	201
Mean/median follow-up time in days (SD│range)	613.6/335 (675.3│8–3,257)
Mean/median time from first observable EC treatment until index date in days (SD│range)	396.5/283 (367.1│91–3,085)
Main characteristics assessed in the fixed 12-month pre-index period	
Mean/median age in years (SD│range)	68.9/71.0 (9.2│35–86)
Mean CCI (SD│range)	9.3 (2.7│2–18)
*N* (%) patients documented with another primary malignant neoplasm	177 (88.1)
Location	
Other female genital organs	134 (66.7)
Mesothelial and soft tissue	35 (17.4)
Breast	32 (15.9)
Digestive organs	24 (11.9)
Respiratory and intrathoracic organs	19 (9.5)
Other specified sites (N < 10 patients)	23 (9.5)
*N* (%) patients documented with a secondary malignant neoplasm	170 (84.2)
Location	
Respiratory or digestive organs nodes	138 (68.7)
Lymph	82 (40.8)
Other or unspecified sites	77 (38.3)
*N* (%) patients with MSI-H/dMMR test	12 (6.0)

*CCI* Charlson Comorbidity Index, *dMMR* defect in mismatch repair gene, *EC* endometrial cancer, *MSI-H* high levels of microsatellite instability, *SD* standard deviation

Cases aged below 18 years and cases without continuous insurance coverage during the period between January 1, 2010, and the index date were excluded from the analysis sample. Finally, all patients in the analysis sample should have had data available for a minimum follow-up of 12 months or until death, whichever came first.

### Analysis periods

Identified patients were observed in a longitudinal analysis to observe OS, treatment patterns, and potential treatment changes over time, starting with the date of the first post-platinum-based treatment (index date). The observation ended at the patient’s death, the end of insurance, or the end of data availability (June 30, 2020).

A fixed 12-month pre-index period (baseline) was used to describe patient characteristics at the time of the post-platinum-based therapy. Additionally, a patient individual pre-index period covering the entire available period between the first-ever observed EC-related treatment start until the index date (modified baseline period) was considered in order to describe the treatment history.

### Outcomes and analyses

#### Baseline characteristics

Besides age, patients’ baseline profiles were focused on comorbidity status. The burden of specific comorbidities and the presence of other malignancies were assessed based on confirmed outpatient diagnoses and inpatient diagnoses documented during the 12-month pre-index period. One diagnosis was sufficient to define a patient as having the comorbidity/disease. Based on the diseases identified, the overall comorbidity status was expressed by calculating the Charlson Comorbidity Index (CCI) (Chae et al. 2011). Additionally, the proportion of patients who were tested for high levels of microsatellite instability (MSI-H) or defect in a mismatch repair gene (dMMR) within a period from 3 months before until 12 months after the index date was assessed.

Furthermore, the treatment history from the first observed EC-related treatment start until the index date (modified baseline period) was described, based on inpatient and outpatient care and outpatient prescriptions.

For all categorical variables, the number and percentage of patients in each category were reported. Summary statistics, including mean, standard deviation (SD), and range, were applied for all continuous variables.

#### Overall survival

All-cause OS after the start of the first post-platinum-based treatment (index date) was assessed by Kaplan–Meier analysis. Failure was defined as the date of all-cause death, with observations censored when insurance coverage ended or the end of data availability (June 30, 2020) was reached. Based on Kaplan–Meier estimates, the median survival time and the proportion of patients who survived 3, 6, 12, and 24 months were reported.

#### EC-related post-platinum-based treatments

The real-world treatment pattern was described for the patient individual post-index period after the start of a post-platinum-based therapy.

Hysterectomy, lymphadenectomy, and radiotherapy (including external radiation therapy and brachytherapy) were considered as EC-related nonpharmacological treatments (inpatient and outpatient procedure codes used are provided in Supplemental Table 1).

EC-related pharmacological treatments were identified by considering inpatient procedures and outpatient prescriptions (Supplemental Table 1). Due to the German reimbursement system for hospitalizations, for inpatient chemotherapy procedures the identification of specific substances is only possible to a limited extent. Therefore, the treatment analysis includes specific agents as well as inpatient chemotherapy procedures in parallel.

As treatment lines are not explicitly captured in the claims data, an algorithm based on prescription/procedure dates to classify treatment episodes was implemented. The first line of post-platinum treatment (LOT) was defined as the first prescription or first inpatient treatment with an EC-related agent at least 3 months after the platinum-based therapy. All agents prescribed or inpatient treatments given within the first 3 months of starting a LOT were considered part of combination therapy. Any new agent prescribed/applied more than 3 months after initiating the LOT was defined as the start of a new LOT. A LOT was assumed discontinued at the start of a new LOT or in case of a gap between the prescriptions/administrations of the drugs belonging to the respective LOT of more than 3 months. If the same agent was prescribed/administrated again after a gap of more than 3 months (“re-start”), this was also defined as a new LOT. The 3-month gap definition was supported by sensitivity analyses where smaller and larger time-gaps of at least 1 month and at least 6 months between the start of platinum-based treatment and the next LOT were assessed. The 3-month gap definition also reflects the mean duration of cycle length and frequency of different commonly used anticancer interventions (Brooks et al. 2019; Johnson et al. 2011) and the commonly used imaging test intervals in the advanced setting.

Descriptive statistics were applied to report the frequency of different agents and treatment combinations observed in each LOT. Furthermore, the time to start of a second treatment line or death after initiating the first post-platinum-based therapy was evaluated by means of Kaplan–Meier analysis and visualized by Kaplan–Meier curves. The observations were censored in case of end of insurance coverage or end of data availability (June 30, 2020).

#### Healthcare resource utilization

HCRU was investigated in terms of the number of outpatient visits (specialists and general practitioners [GPs], approximated by counted dates of invoiced codes according to the EBM), the number of inpatient visits, and the number of days in the hospital. Both all-cause and EC-related utilization were assessed. For outpatient physician visits, cases were considered EC-related if any diagnosis was coded as EC (ICD-10-GM code C54.-, excluding C54.2). Hospitalizations were defined to be EC-related if the associated main diagnosis was EC.

The HCRU items were assessed for the fixed 12-month pre-index period (baseline), the first 12 months after index, and the entire patient-individual follow-up period after the start of the post-platinum-based therapy. Generally, all items were reported per observed patient-year. In addition, the frequency of patients with at least 1 visit/hospitalization and the mean length of stay of all observed hospitalizations were reported.

#### Regulatory aspects and general considerations

As the study addressed a retrospective anonymized dataset, no ethical review and no informed consent of patients were needed. However, the study protocol was reviewed by a scientific steering committee and the data owner. The work on the dataset conformed to all social security data protection requirements.

Statistical analyses were performed using Microsoft SQL Server 2014 (Microsoft Corporation, Redmond, WA), STATA/MP 14 (StataCorp LLC, College Station, TX), and Microsoft Excel.

## Results

### Patient selection and baseline characteristics

**Fig. 1:**
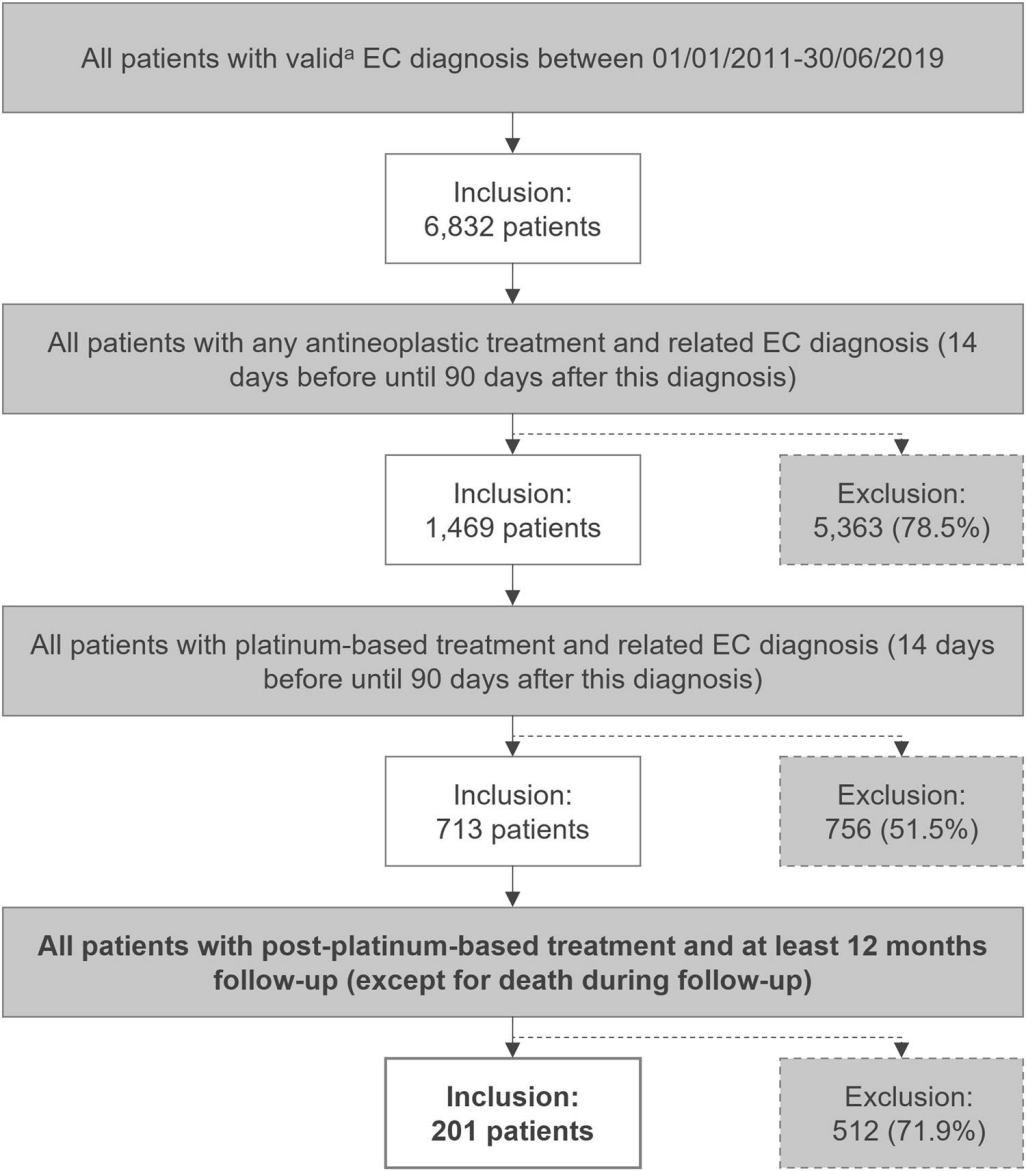
Attrition chart for the patient selection EC, endometrial cancer. ^a^At least one inpatient EC diagnosis (ICD-10-GM code C54.-, excluding C54.2 [malignant neoplasms of myometrium]) or two confirmed outpatient EC diagnoses made by a specialist

Of the 6,832 identified patients with a valid EC diagnosis between January 01, 2011 and June 30, 2019, 1,469 had received anticancer treatment and, of those, 713 had received a platinum-based treatment. Out of these, 201 patients with post-platinum-based anticancer treatment (definition of EC-related treatments and identification codes are provided in Supplemental Table 1) and a minimum follow-up of 12 months were identified (Fig. 1). The majority of the excluded patients did not receive any anticancer treatment (5,363 patients). The mean patient individual follow-up was 613.6 days (range: 8–3,257 days), and the average duration between the first observable EC-related treatment in the pre-index period and the index date was 396.5 days (range: 91–3,085 days, Table 1). In 201 patients included in the analysis sample, the mean age was 68.9 years (SD: 9.2, Table 1) and the mean CCI was 9.3 (SD: 2.7). Within this analysis sample, 177 (88.1%) and 170 (84.2%) patients had another documented primary or secondary malignancy, respectively. In total, 12 patients (6.0%) were tested for MSI-H or dMMR within the period from 3 months before until 12 months after the index date.

### Overall survival

**Fig. 2:**
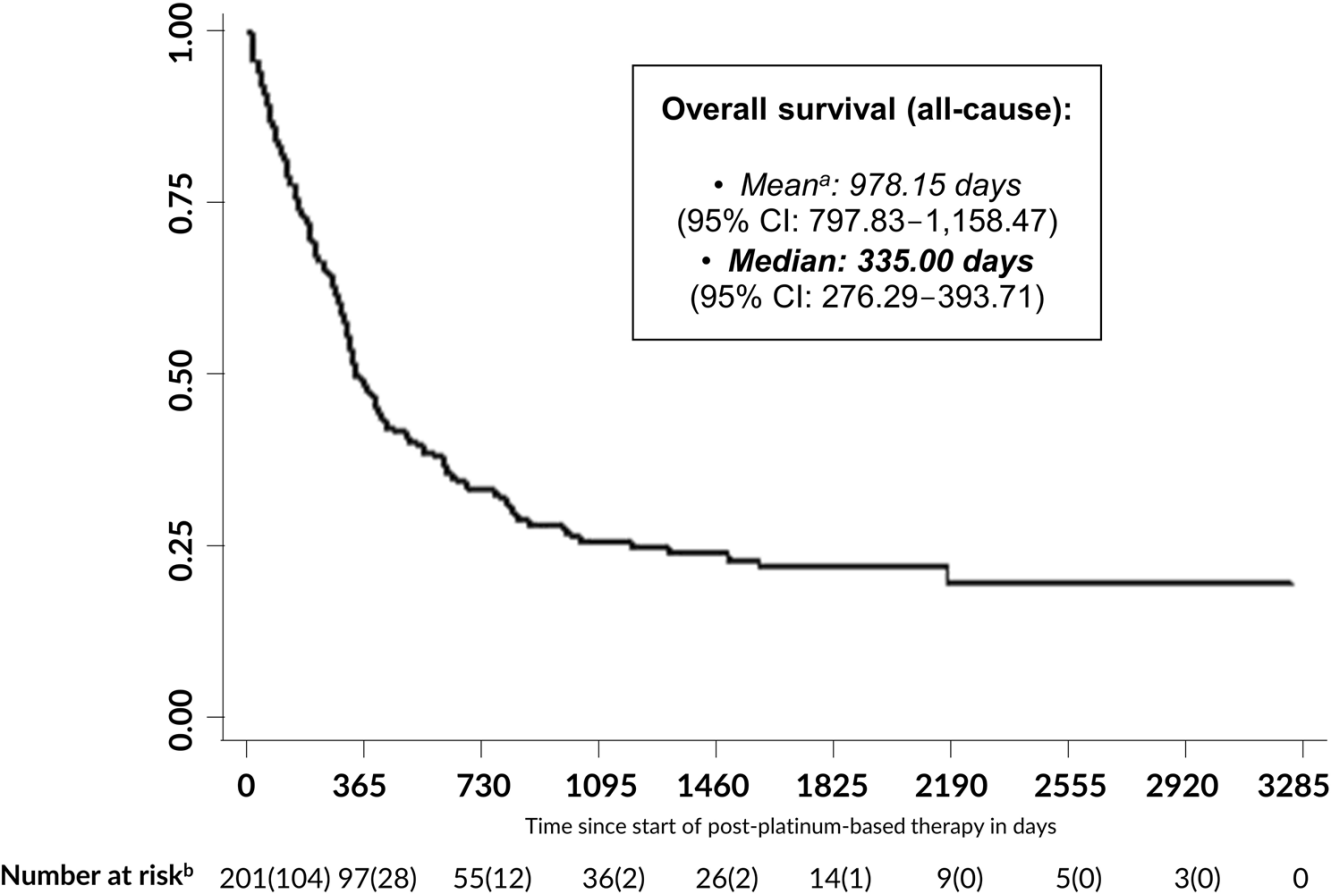
Kaplan–Meier all-cause overall survival curve *CI* confidence interval ^a^Mean = restricted mean. Estimation is limited to the largest survival time if it is not censored. ^b^Number at risk: *N* (deaths in period)

Median OS in the analyzed patient population was 335.0 days (95% confidence interval [CI]: 276.29–393.71; Fig. 2). After 3, 6, 12, and 24 months, the estimated survival rates were 84.1%, 72.6%, 48.3%, and 33.2%, respectively.

### EC-related treatments

#### Modified baseline period

**Fig. 3:**
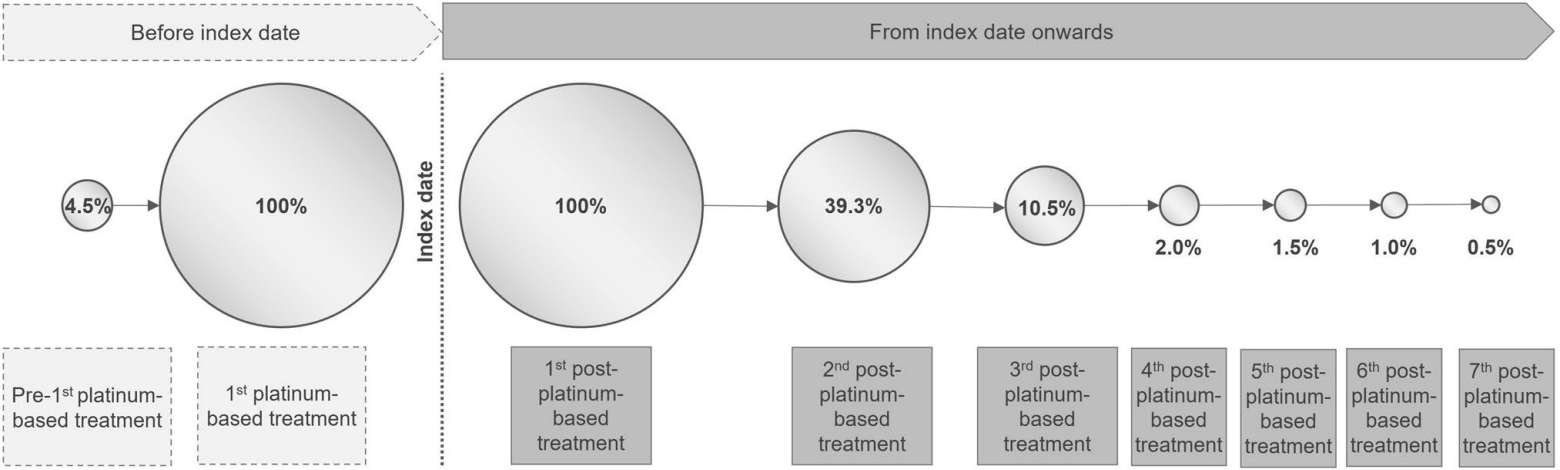
Pharmaceutical treatment during the entire observational period

EC treatment was analyzed in the modified baseline period between the first-ever observed EC-related treatment start until index date. For nine patients (4.5%), we identified at least one treatment before the platinum-based treatment (Fig. 3): Four patients received endocrine therapy, and five patients received other anticancer treatment combinations.

All 201 patients received a first platinum-based treatment in the baseline period. The most frequently observed first platinum-based treatment regimen was carboplatin and paclitaxel (partly combined with additional agents) in 90 patients (44.8%), followed by inpatient chemotherapy procedures without any specific information on prescribed chemotherapy agents in 86 patients (42.8%). Other carboplatin combinations were received by 14 patients (7.0%); cisplatin combinations were given to 10 patients (5.0%); one patient received oxaliplatin (0.5%) (Table 2).

**Table 2:** First observed platinum-based treatment (before index treatment)

Observed agents/procedures	*N* (%) patients (*N* = 201)
Paclitaxel	109 (54.3%)
Carboplatin	104 (51.7%)
Noncomplex chemotherapy (inpatient)	82 (40.8%)
Moderate complex chemotherapy (inpatient)	42 (20.9%)
Doxorubicin^a^	14 (7.0%)
Bevacizumab	13 (6.5%)
Cisplatin	11 (5.5%)
23 other agents/procedures (< 10 patients)	37 (18.4%)
**Observed regimen**	
Carboplatin and paclitaxel (and combinations)	
Inpatient procedures	
Other carboplatin combinations	
Cisplatin combinations	
Oxaliplatin combination	

^a^Doxorubicin and liposomal doxorubicin data were captured separately but were not distinguished between in these analyses

Before start of the first post-platinum-based treatment, 13.4% of the observed patients had a surgery (*N* = 27; hysterectomy and/or lymphadenectomy) and 22.9% had a radiotherapy (*N* = 46).

### Follow-up period

After start of the post-platinum-based treatment, seven patients underwent a hysterectomy and/or lymphadenectomy (3.5%), and 36 patients received radiotherapy (17.9%). Of the 201 patients with a first post-platinum-based LOT, 79 patients (39.3%) received a second LOT. Three or more treatment lines were seen in 21 patients (10.4%). Kaplan–Meier estimation for the median time from first to second post-platinum LOT or death can be found in Supplemental Fig. 1.

The three most frequently observed anticancer agents in the first post-platinum-based LOT were carboplatin (42 patients, 20.9%), paclitaxel (33 patients, 16.4%), and doxorubicin (30 patients, 14.9%). The treatment regimens varied from different inpatient chemotherapy procedures (11.9% noncomplex; 3.5% moderate complex), endocrine therapy (8.0% medroxyprogesterone; 3.5% tamoxifen), and carboplatin combinations (5.5% with paclitaxel; 3.5% with doxorubicin) to monotherapy options (7.0% doxorubicin; 4.0% paclitaxel). An additional 65 combinations were identified, each being administered to fewer than seven patients (Table 3).

**Table 3:** Observed first-line treatments in the follow-up period

Frequency of observed treatments in the first LOT following platinum-based therapy based on 201 patients analyzed, *N* (%)	
**Observed agents/procedures**	
Carboplatin	42 (20.9%)
Paclitaxel	33 (16.4%)
Doxorubicin^a^	30 (14.9%)
Noncomplex chemotherapy with 2 agents (inpatient)	26 (12.9%)
Medroxyprogesterone	20 (10.0%)
Gemcitabine	17 (8.5%)
Bevacizumab	16 (8.0%)
33 other agents/procedures (each ≤ 10 patients)	77 (38.3%)
**Observed regimen**	
Noncomplex chemotherapy (inpatient)	24 (11.9%)
Medroxyprogesterone (monotherapy)	16 (8.0%)
Doxorubicin (monotherapy)^a^	14 (7.0%)
Carboplatin and paclitaxel	11 (5.5%)
Paclitaxel (monotherapy)	8 (4.0%)
Tamoxifen (monotherapy)	7 (3.5%)
Carboplatin and doxorubicin	7 (3.5%)
Moderate complex chemotherapy (inpatient)	7 (3.5%)
65 other combinations (each < 7 patients)	107 (53.2%)

*LOT* line of treatment

^a^Doxorubicin and liposomal doxorubicin data were captured separately but were not distinguished between in these analyses

In the 79 patients starting a second LOT, carboplatin and paclitaxel were identified as the most frequently prescribed second-line agents (each 20.3%). In total, 30 different agents and procedures in 45 different treatment regimens were seen in the second LOT (Table 4), of which medroxyprogesterone acetate, megestrol acetate, cisplatin, and doxorubicin are approved for the EC indication in Germany.

**Table 4:** Observed second-line treatments in the follow-up period

Frequency of observed treatments in the second LOT following platinum-based therapy based on 79 patients analyzed, *N* (%)	
**Observed agents/procedures**	
Paclitaxel	16 (20.3%)
Carboplatin	16 (20.3%)
Medroxyprogesterone^a^	10 (12.7%)
Doxorubicin^a,b^	8 (10.1%)
Gemcitabine	7 (8.9%)
Tamoxifen	6 (7.6%)
Megestrol^a^	6 (7.6%)
Trabectedin	6 (7.6%)
22 other agents/procedures (< 6 patients)	36 (45.6%)
**Observed regimen**	
Medroxyprogesterone (monotherapy)	7 (8.9%)
Carboplatin and paclitaxel	6 (7.6%)
Doxorubicin (monotherapy)^b^	6 (7.6%)
42 other combinations (< 6 patients)	60 (75.9%)

*EC* endometrial cancer, *LOT* line of treatment

^a^Approved for the EC indication in Germany; ^b^Doxorubicin and liposomal doxorubicin data were captured separately but were not distinguished between in these analyses

### HCRU

In the 12 months before starting the post-platinum-based therapy, most patients visited a GP at least once (180 patients, 89.6%), with 4.4 all-cause and 2.3 EC-related visits per observed patient-year (Table 5). A gynecologist was seen at least once by 121 patients (60.2%), with the majority of the visits being EC-related (1.8 out of 2.4 visits per patient-year). Outpatient oncologist visits were observed less frequently (0.8/0.6 visits/EC-related visits per patient-year). Generally, outpatient care utilization did not change dramatically after the start of the post-platinum-based therapy. The number of gynecologist visits per patient-year increased to 3.4 in the first 12 months of follow-up but was equal to the number observed at baseline when considering the entire follow-up period (Table 5).

**Table 5:** HCRU during baseline and follow-up period

	12-month pre-index period (baseline)	12-month follow-up after initiation of post-platinum-based treatment	Entire follow-up period after initiation of post-platinum-based treatment
*N*	201	201	201
Observed patient-years (py)	201	146.1	337.9
**Outpatient care**			
Any GP visits (all-cause)			
Number (%) patients with at least one visit	180 (89.6%)	201 (100%)	180 (89.6%)
Visits per py	4.4	4.6	4.5
EC-related GP visits			
Number (%) patients with at least one visit	118 (58.7%)	124 (61.7%)	116 (57.7%)
Visits per py	2.3	2.0	2.3
Any gynecologist visits (all-cause)			
Number (%) patients with at least one visit	121 (60.2%)	173 (86.1%)	116 (57.7%)
Visits per py	2.4	3.4	2.4
EC-related gynecologist visits			
Number (%) patients with at least one visit	100 (49.8%)	145 (72.1%)	96 (47.8%)
Visits per py	1.8	2.6	1.8
Any oncologist visits (all-cause)			
Number (%) patients with at least one visit	56 (27.9%)	40 (19.9%)	54 (26.9%)
Visits per py	0.8	0.5	1.0
EC-related oncologist visits			
Number (%) patients with at least one visit	39 (19.4%)	26 (12.9%)	38 (18.9%)
Visits per py	0.6	0.3	0.7
Visits at any other specialist (all-cause)			
Number (%) patients with at least one visit	160 (79.6%)	185 (92.0%)	156 (77.6%)
Visits per py	4.5	4.4	4.3
EC-related visits at any other specialist			
Number (%) patients with at least one visit	73 (36.3%)	79 (39.3%)	61 (30.3%)
Visits per py	0.8	0.8	0.9
**Inpatient care**			
Any hospitalizations (all-cause)			
Number (%) patients with at least one hospitalization	183 (91.0%)	190 (94.5%)	179 (89.1%)
Hospitalizations per py	4.6	3.3	5.2
Inpatient days per py	33.3	23.9	39.6
Mean (SD) length of hospitalizations^a^	7.3 (10.4)	7.2 (11.6)	7.6 (11.8)
EC-related hospitalizations			
Number (%) patients with at least one hospitalization	119 (59.2%)	120 (59.7%)	108 (53.7%)
Hospitalizations per py	1.9	1.4	2.3
Inpatient days per py	16.0	8.5	17.2
Mean (SD) length of hospitalizations^a^	8.5 (13.0)	6.2 (12.0)	7.4 (13.3)

*EC* endometrial cancer, *GP* general practitioner, *HCRU* healthcare resource utilization, *py* patient-year, *SD* standard deviation

^a^Average length of stay of all observed hospitalizations

The hospitalization rate in the analyzed patient sample was generally high. In the 12-month pre-index period, 183 patients (91.0%) had at least one inpatient stay. In total, 4.6 hospitalizations and 33.3 inpatient days per patient-year were observed in the baseline period, with 1.9/16.0 of these hospitalizations/inpatient days being related to EC. The number of hospitalizations per patient-year decreased to 3.3 (EC-related: 1.4) in the first 12 months of follow-up and increased to 5.2 (EC-related: 2.3) in the entire follow-up period. Correspondingly, the number of inpatient days per patient-year in the first 12 months after the start of post-platinum-based therapy was lower compared to baseline (23.9 days per patient-year, with 8.5 days being related to EC) but rose to 39.6 days per patient-year (EC-related: 17.2 days) in the entire follow-up period.

The mean length of stay for an EC-related hospitalization was slightly longer than the average reported for all hospitalizations (8.5 vs 7.3 days) in the 12-month pre-index period, but marginally shorter when considering mean length of stay for all hospitalizations in the first 12 months after the start of the post-platinum-based therapy (6.2 vs 7.2 days; Table 5).

## Discussion

Based on a large German claims dataset, this study evaluated the real-world treatment of patients with recurrent or advanced EC treated with an anticancer pharmacological treatment after platinum-based chemotherapy. The main strength of this analysis is the use of a large, unselected database that provides complete information on mortality and outpatient/inpatient treatment of patients.

Based on the predefined selection criteria, 201 patients with post-platinum-based treatment initiation were identified. The investigated patients had a very high comorbidity burden indicated by a mean CCI of 9.3, and most patients had another documented primary and/or secondary malignancy. The median OS was less than 1 year, which is generally in line with the findings of other investigations with comparable populations (Cosgrove et al. 2021; Ueda et al. 2011).

The most frequently observed first-line post-platinum-based agents were carboplatin, paclitaxel, and doxorubicin. In total, 40 different agents resulting in more than 70 combinations were identified. Initiation of a second LOT after platinum-based chemotherapy was seen in 79 patients. The distribution of agents used was similar to the first LOT, with paclitaxel and carboplatin again being the most frequently observed agents. The variety of treatment combinations identified in the second LOT was also comparable to the first LOT. Even if the co-occurrence of other malignancies influences treatment decisions, the high number of different therapy regimens seen in routine clinical practice emphasizes the urgent need for more clear and targeted strategies in the recurrent and advanced EC setting.

New targeted treatment options are becoming available. These treatment options require the assessment of the molecular subtype, which is not mentioned in the current German guideline (Emons and Steiner 2018) but already suggested in the new ESGO guideline (Concin et al. 2021). Genetic tumor testing is therefore not part of the current care routine of patients with EC in Germany. In the analyzed sample, an MSI-H/dMMR test was conducted in only 6% of the patients. Based on our analysis, immune checkpoint programmed cell death protein-1 inhibitors were rarely used as EC treatment in clinical practice due to lack of approval during the observation period. We found only one patient with advanced EC receiving pembrolizumab (as first-line treatment); however, the treatment landscape is changing as two checkpoint inhibitor is now approved for MSI-H/dMMR patients. As a result of this approval, testing rates for MSI-H/dMMR will increase. Additionally the European Medicines Agency recently approved the pembrolizumab monotherapy and in combination with lenvatinib for patients with advanced or recurrent EC (European Medicines Agency 2021).

A high hospitalization rate was found in observed patients, which corresponds with the comorbidity profile of the patients and the advanced stage of their disease. On average, a patient in the analysis sample spent 39.5 days a year in hospital, with 17.2 days related to hospitalizations with EC as the main diagnosis. Initially, the number of inpatient days per patient-year in the first 12 months after the start of post-platinum-based therapy was lower compared to baseline; however, this rose to 39.6 days per patient-year, most likely attributed to increases in hospitalization prior to death and the high mortality rate.

Generally, the nature of the claims dataset used for this analysis precludes selection bias and facilitates generalizability. Nevertheless, some limitations should be acknowledged. One limitation of this study is the limited sample size, which affects the generalizability of our results. However, it needs to be considered that even though EC is a common cancer, advanced and recurrent disease is rare, and sample sizes above 200 are rarely achieved in other studies. The number of patients with a valid EC diagnosis, and the percentage who received anti-neoplastic agents and progressed to post-platinum treatment appear plausible based on the following: recent estimates of endometrial cancer incidence in Germany (Zentrum für krebsregisterdaten 2021), most patients with EC are diagnosed at an early stage and do not receive antineoplastic treatment (Colombo et al. 2016), and German treatment guidelines do not specifically recommend platinum-based regimens as chemotherapy for recurrent EC (Emons and Steiner 2018). Another general limitation of this analyses based on German claims data is the lack of clinical data available. Thus, the methodology employed in our study allows no detailed consideration of factors influencing patient individual treatment decisions (for example, existing contraindications, the entire clinical situation with co-malignancies, treatment failure, or the occurrence of adverse events). Furthermore, information on disease stage and treatment lines could only be derived by proxies. Finally, by using only data from one regional German sickness fund, the results might not be fully representative of the whole German population, although treatment patterns and reimbursement rules for statutory health insurances are comparable across Germany (Ghiani et al. 2022; Hardtstock et al. 2020a, b).

## Conclusions

Our study showed that a wide variety of therapy regimes are used in patients in Germany who progressed after platinum-based therapy, confirming the lack of a clear therapeutic strategy for this patient population. The poor prognosis of these patients highlights the urgent need for new treatment strategies.

## Supplementary Information

Below is the link to the electronic supplementary material.

Supplementary file1 (DOCX 187 KB)

Supplementary file2 (PDF 201 KB)

## Data Availability

GSK makes available anonymized individual participant data and associated documents from interventional clinical studies which evaluate medicines, upon approval of proposals submitted to www.clinicalstudydatarequest.com. To access data for other types of GSK sponsored research, for study documents without patient-level data, and for clinical studies not listed, please submit an enquiry via the website.
