# Supplementary material for: Real-world treatment of German patients with recurrent and advanced endometrial cancer with a post-platinum treatment: a retrospective claims data analysis
Source: J Cancer Res Clin Oncol. 2022 Jul 16;149(5):1929–39. doi: 10.1007/s00432-022-04183-y (PMC10097742; doi:10.1007/s00432-022-04183-y)
Supplement: Supplementary file 1 — Supplementary file1 (DOCX 187 KB) [file 432_2022_4183_MOESM1_ESM.docx]

# Supplementary Information

**Real-world treatment of German patients with recurrent and advanced endometrial cancer after an initial platinum-based treatment: A retrospective claims data analysis**

*Journal of Cancer Research and Clinical Oncology*

Antje Mevius^1^, Florian Karl^2^, Margarethe Wacker^2^, Robert Welte^2*^, Stefanie Krenzer2, Theresa Link^3^, Ulf Maywald^4^, Thomas Wilke^1^

^1^IPAM e.V., University of Wismar, Germany; ^2^GlaxoSmithKline GmbH & Co. KG, München, Germany; ^3^Universitätsklinikum Carl Gustav Carus an der Technischen Universität Dresden, Germany; ^4^AOK PLUS, Dresden, Germany

*Affiliation at time of study

Corresponding author: Antje Mevius, antje.mevius@ipam-wismar.de, Tel. 0049 3841 7581020

## Supplemental Table 1 Definition of EC-related treatments

| **Treatment** | **Identification codes** |
| --- | --- |
| Platinum-based treatment | - ATC code L01XA- (outpatient setting) - OPS code 8-54- (inpatient chemotherapy) |
| Any anticancer treatment | - ATC codes L01- (outpatient chemotherapy) - ATC codes L02AB-/L02BA01/L02BG- (endocrine therapy) - OPS code 8-54- (inpatient chemotherapy) |
| Hysterectomy | OPS codes 5-682/5-683/5-685 |
| Lymphadenectomy | OPS codes 5-401/5-402/5-403/5-404/5-406/5-407 |
| Radiotherapy | - EBM codes 25321/25332 (outpatient procedure) - OPS code 8-52/8-53 (inpatient procedure) |
| ATC, Anatomical Therapeutic Chemical classification; EBM, German Uniform Valuation Scheme; EC, endometrial cancer; OPS, Operationen- und Prozedurenschlüssel | |

## Supplemental Fig. 1 Time from first to second post-platinum LOT or death (composite)


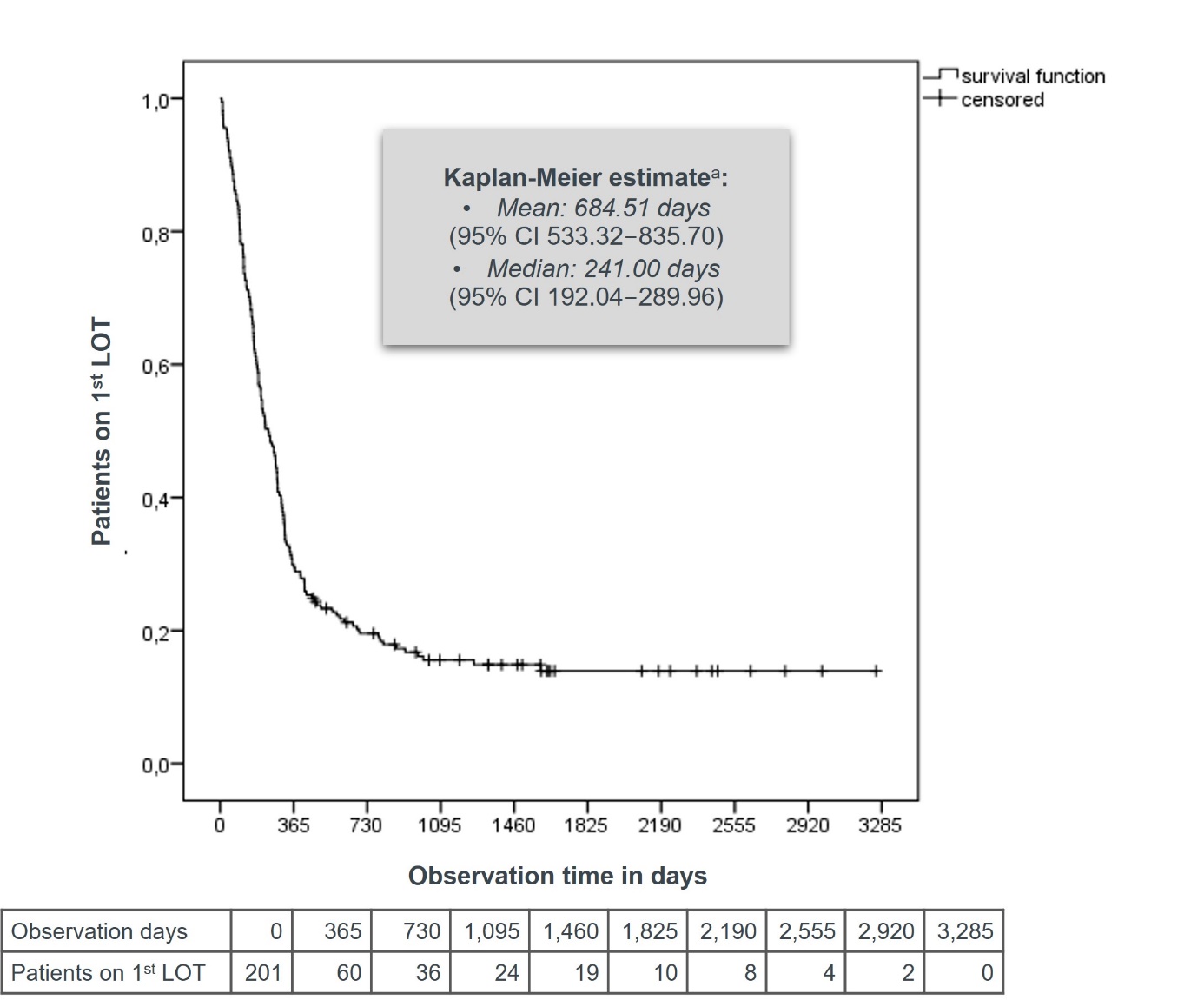


CI, confidence interval; LOT, line of treatment

^a^Censored for 1^st^ line treatment until end of observation (including a 90-day gap)
