# Supplementary figures and images for: Real-world treatment of German patients with recurrent and advanced endometrial cancer with a post-platinum treatment: a retrospective claims data analysis
Source: J Cancer Res Clin Oncol. 2022 Jul 16;149(5):1929–39. doi: 10.1007/s00432-022-04183-y (PMC10097742; doi:10.1007/s00432-022-04183-y)

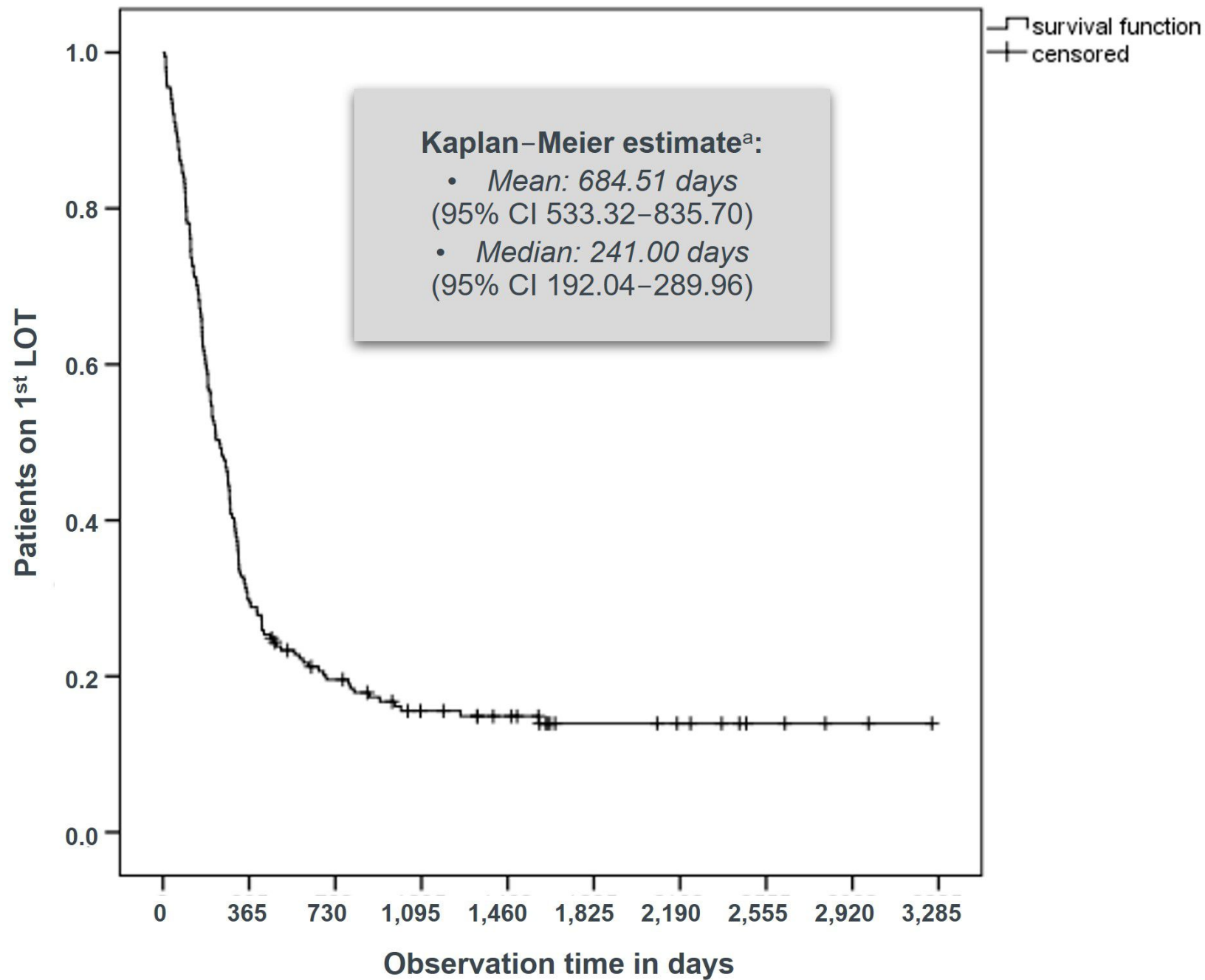

| Observation days                | 0   | 365 | 730 | 1,095 | 1,460 | 1,825 | 2,190 | 2,555 | 2,920 | 3,285 |
|---------------------------------|-----|-----|-----|-------|-------|-------|-------|-------|-------|-------|
| Patients on 1 <sup>st</sup> LOT | 201 | 60  | 36  | 24    | 19    | 10    | 8     | 4     | 2     | 0     |

Supplement: Supplementary file 2 — Supplementary file2 (PDF 201 KB) [file 432_2022_4183_MOESM2_ESM.pdf]
